# Study of hydrogen sulfide biosynthesis in synovial tissue from diabetes-associated osteoarthritis and its influence on macrophage phenotype and abundance

**DOI:** 10.1007/s13105-023-00968-y

**Published:** 2023-06-19

**Authors:** Natalia Lendoiro-Cino, Arianna Rodríguez-Coello, Anna Saborido, Elena F-Burguera, Jennifer A. Fernández-Rodríguez, Rosa Meijide-Faílde, Francisco J. Blanco, Carlos Vaamonde-García

**Affiliations:** 1 Grupo de Investigación en Reumatología, Instituto de Investigación Biomédica de A Coruña (INIBIC), Complexo Hospitalario Universitario de A Coruña (CHUAC), Sergas, Universidade da Coruña (UDC), 15006 A Coruña, Spain; 2 Centro de Investigación Biomédica en Red de Bioingeniería, Biomateriales y Nanomedicina (CIBER-BBN), Madrid, Spain; 3 Grupo Envejecimiento e Inflamación, Instituto de Investigación Biomédica de A Coruña (INIBIC), Complexo Hospitalario Universitario de A Coruña (CHUAC), Sergas, Universidade da Coruña (UDC), 15006 A Coruña, Spain; 4 Grupo de Terapia Celular y Medicina Regenerativa, Centro Interdisciplinar de Química e Bioloxía (CICA), Departamento de Fisioterapia, Medicina y Ciencias Biomédicas, Facultad de Ciencias da Saúde, Universidade da Coruña (UDC), 15006 A Coruña, Spain; 5 Grupo de Investigación en Reumatología y Salud, Centro Interdisciplinar de Química e Bioloxía (CICA), Departamento de Fisioterapia, Medicina y Ciencias Biomédicas, Facultad de Fisioterapia, Universidade da Coruña (UDC), 15006 A Coruña, Spain; 6 Grupo de Investigación en Reumatología y Salud, Centro Interdisciplinar de Química e Bioloxía (CICA), Departamento de Bioloxía, Facultad de Ciencias, Universidade da Coruña (UDC), 15008 A Coruña, Spain

**Keywords:** Hydrogen sulfide, Osteoarthritis, Type 2 diabetes, Macrophages, Glucose stress, Heme oxygenase-1

## Abstract

Type 2 diabetes (DB) is an independent risk factor for osteoarthritis (OA). However, the mechanisms underlying the connection between both diseases remain unclear. Synovial macrophages from OA patients with DB present a marked pro-inflammatory phenotype. Since hydrogen sulphide (H_2_S) has been previously described to be involved in macrophage polarization, in this study we examined H_2_S biosynthesis in synovial tissue from OA patients with DB, observing a reduction of H_2_S-synthetizing enzymes in this subset of individuals. To elucidate these findings, we detected that differentiated TPH-1 cells to macrophages exposed to high levels of glucose presented a lower expression of H_2_S-synthetizing enzymes and an increased inflammatory response to LPS, showing upregulated expression of markers associated with M1 phenotype (i.e., CD11c, CD86, iNOS, and IL-6) and reduced levels of those related to M2 fate (CD206 and CD163). The co-treatment of the cells with a slow-releasing H_2_S donor, GYY-4137, attenuated the expression of M1 markers, but failed to modulate the levels of M2 indicators. GYY-4137 also reduced HIF-1α expression and upregulated the protein levels of HO-1, suggesting their involvement in the anti-inflammatory effects of H_2_S induction. In addition, we observed that intraarticular administration of H_2_S donor attenuated synovial abundance of CD68^+^ cells, mainly macrophages, in an *in vivo* model of OA. Taken together, the findings of this study seem to reinforce the key role of H_2_S in the M1-like polarization of synovial macrophages associated to OA and specifically its metabolic phenotype, opening new therapeutic perspectives in the management of this pathology.

## Introduction

Osteoarthritis (OA) is the most common chronic rheumatic disease, afflicting over 300 million people worldwide [31]. This pathology can affect any joint, but is predominantly observed in hands, knees, hips, lower back and neck, where it causes articular cartilage degradation together with subchondral bone thickening, osteophyte formation, and synovial inflammation [51]. The main symptoms of OA are joint pain and stiffness, and in turn limited movement of joint. The aetiology of OA is multifactorial and includes joint injury, obesity, aging, and genetic and environmental factors among others [30]. In this regard, growing evidence indicate that type 2 diabetes mellitus (DB) is an independent risk factor for OA that favours its development but also incidence and severity [67, 70].

Molecular mechanisms underlying the link between DB and OA remain to be completely understood, although one of the pathways that are thought to support role of DB on OA pathogenesis is oxidative stress resulting from chronic hyperglycaemia, which in turn leads to overproduction of pro-inflammatory cytokines and activation of catabolic processes in joint tissues [67, 70]. Likewise, although OA was historically known as a “wear and tear” condition and result of gradual degradation of cartilage, nowadays it is widely accepted that inflammation has a critical role in its pathogenesis [50]. The inflammation in OA leads to many pathologic changes in tissues surrounding joint. Thus, OA is characterized by a chronic and low-grade inflammation primarily mediated through the innate immune system [50, 52], which is clearly distinguishable from that observed in other rheumatic diseases like rheumatoid arthritis.

Macrophages are tissue-resident or infiltrated immune cells critical for innate immunity that secrete a wide range of biologically active molecules and play an important role in the initiation, maintenance, and resolution of inflammation [19, 72]. Synovial inflammation, synovitis, observed in OA patients is featured by the infiltration of macrophages, whose increment in number has been correlated with clinical symptoms of the disease [2, 30]. There is a high heterogeneity of macrophages in the OA synovial tissue however, being involved in both catabolic and anabolic pathways in the joint. Notwithstanding different macrophage phenotypes have been proposed in OA [36], they are generally categorized into two broad but distinct subsets as either classically activated M1-like macrophages, mainly involved in pro-inflammatory responses, or alternatively activated M2-like macrophages, mainly involved in anti-inflammatory responses [41, 74]. Likewise, macrophage phenotype could be influenced by the microenvironment in the inflamed joint, as well as the stage and endotype of the disease [36]. In this regard, accumulating evidence supports a crucial role of macrophage polarization in the development of DB and thus, an increment of M1-like macrophages has been associated with inflammation in this subset of patients [41]. In agreement, different studies have observed that synovium from OA patients with DB noticeably contains more macrophages and shows elevated levels of pro-inflammatory mediators in comparison to the synovium from OA patients without DB [16, 18].

Hydrogen sulphide (H_2_S) is small gaseous mediator involved in several physiological and pathological processes [12]. For instance, it has been described that induction of H_2_S production is a protective mechanism against glucotoxicity-induced oxidative stress [69]. H_2_S is produced endogenously from l-cysteine by two pyridoxal-50-phosphate-dependent enzymes, cystathionine β-synthase (CBS) and cystathionine γ-lyase (CSE), and to a lower extent, by 3-mercaptopyruvate sulphurtransferase (MPST) [6, 47]. Different studies have observed that joint tissues are able to synthetise these enzymes and produced H_2_S [4, 12, 46], showing anti-inflammatory and anti-oxidant effects in joint cells in *in vitro* and *in vivo* models of OA [5, 12, 63]. However, cartilage from both OA and OA-DB patients show an impairment in the expression of these H_2_S-synthesizing enzymes [4, 46]. H_2_S levels are likewise reduced in DB and thus evidence suggest that this event could underlie DB pathogenesis and associated complications [44, 61], likely due to the fact that endogenous level of the gas has been linked to macrophage activation and polarization, regulating the balance of M1/M2-like macrophage by reducing M1 abundance in adipose tissue from DB patients [59, 78].

Nonetheless, despite the fact that growing evidence suggest a reduction of H_2_S in synovium from DB patients could assist in macrophage polarization towards a pro-inflammatory phenotype favouring OA pathogenesis, the expression of H_2_S synthesizing enzymes in the synovial tissue from this subset of patients, as well as the beneficial effect of H_2_S induction on M1/M2-like macrophage balance, have yet to be investigated. For this purpose, we compared the expression of CSE and CBS in synovium from OA and OA-DB patients and evaluated the effect of a slow-releasing donor of H_2_S on macrophage polarization in TPH-1 cells differentiated into macrophage under an *in vitro* model of diabetes-associated glucotoxicity.

## Material & methods

### Human samples and cell culture

Synovial tissues were obtained from 10 OA patients (4 females and 6 males; median age was 73.3 [77.5–69.5] years old) and 10 OA-DB patients (2 females and 8 males; median age was 74.6 [83.4–65.8] years old) who underwent joint replacement surgery and gave informed consent. This study was reviewed and approved by the Local Ethics Committee. Samples were subsequently embedded in paraffin for obtaining 4 µm-thick histological section. For *in vitro* experiments, we used the immortalized cell line of monocytes TPH-1 that was maintained in Roswell Park Memorial Institute Medium (RPMI)-1640 (ThermoFisher, Madrid, Spain) containing 10% fetal bovine serum (FBS), 2 mM l-glutamine, 100 mg/mL streptomycin, and 100 U/mL penicillin (Lonza, Basel, Switzerland). TPH-1 were differentiated into macrophages after treatment with phorbol-12-myristate-13-acetate (PMA; 500 nM) (Sigma-Aldrich, St Lois, USA) for 3 h [8]. Thereafter, macrophages were incubated in RPMI-1640 2% FBS with 1 g/l glucose (normal glucose, NG) or 4.5 g/l (high glucose, HG) and stimulated with bacterial lipopolysaccharides (LPS; 1 ug/ml) (Sigma-Aldrich, San Luis, MO, USA), a classical inductor of M1-like macrophages [14, 29], in the presence or absence of a slow-releasing H_2_S donor, GYY-4137 (500 µM) (SantaCruz Biotechnology, Heidelberg, Germany), based on previous research [46, 66].

### Rat samples of synovial tissue

Samples were obtained from an *in vivo* model of OA previously performed by our group [63]. Briefly, experimental OA was induced by the transection of the medial collateral ligament and the removal of the medial meniscus of the left joint, and the right joint was sham-operated and employed as a control. Then, the animals were randomized into two groups (3 rats per group): OA, control injection, injected intraarticularly with vehicle, i.e. saline; and OA-GYY, sulphide injection, treated with a single intraarticular injection of the slow-releasing H2S donor GYY-4137 [63]. Single injection was carried out at day 7 after surgery. Animals were euthanized at day 40. All the animal experiments were performed according to protocols approved by the Local Ethical Committee of Animal Experimentation (Comité de Ética de Experimentación Animal de la Xerencia de Xestión Integrada A Coruña (CEEA-XXIAC); 15002/2015/12) and European Directive 2010/63, including treatment with antibiotics, analgesics, and painkiller drugs when corresponded.

### Gene expression analysis by real-time PCR

Cells were stimulated as previously indicated for 24 h and total RNA was extracted and purified using TRIzol Reagent (Invitrogen, Paisley, UK), chloroform (Sigma-Aldrich), and isopropanol (Sigma-Aldrich) following the manufacturer’s recommendations. The NZY First-Strand cDNA Synthesis Kit (Nzytech, Lisboa, Portugal) was used to obtain complementary DNA (cDNA). Reverse transcription of 1000 ng of RNA from each sample was performed in a 96-Well Thermal Cycler (Applied Biosystems, Thermo Fisher Scientific, Madrid, Spain). cDNA obtained was then amplified by quantitative real-time PCR using the Fast SYBR™ Green master mix (Roche Diagnostics, Abingdon, UK) in a LightCycler 480 instrument (Roche Diagnostics), employing the primers shown in Table 1. Relative gene expression was calculated with the 2 − ΔΔCT method. Hypoxanthine phosphoribosyltransferase 1 (HPRT) was employed as the reference gene for normalization. All primers were purchased from Invitrogen.

**Table 1:** Primer sequences used for real-time qPCR assays

Gene	NCBI accession number	Forward primer (5’-3’)	Reverse primer (5’-3’)
CBS	NM_000071.3	AGGAGAAGTGTCCTGGATGC	TAGGTTGTCTGCTCCGTCTG
CSE	NM_001902.6	GCATTTCAAAAACGGAATGG	CTCATGCTGTGGATGAGAGG
CD11c	NM_000887.5	TGACATTGCATCGAAGCCCTC	TCCGTACCCTCAATGGCAAAG
CD86	NM_175862.5	AGACCTGCCATGCCAATTTG	CGAATCAAAACTTGTGCGGC
CD163	NM_004244.6	TCACAATGAAGATGCTGGCG	CCTGCAAACCACATCAGCTT
CD206	NM_002438.4	AGCCAACACCAGCTCCTCAAGA	CAAAACGCTCGCGCATTGTCCA
HPRT	NM_000194.3	TGATAGATCCATTCCTATGACTGTAGA	CAAGACATTCTTTCCAGTTAAAGTTG
IL-6	NM_000600.5	GATGAGTACAAAAGTCCTGATCCA	CTGCAGCCACTGGTTCTGT
IL-8	NM 000584.3	GAGCACTCCATAAGGCACAAA	ATGGTTCCTTCCGGTGGT
iNOS	NM_000625.4	GCTGCCAAGCTGAAATTGA	GATAGCGCTTCTGGCTCTTG

*CBS* cystathionine β-synthase; *CSE* cystathionine γ-lyase; *CD* cluster of differentiation; *HPRT* Hypoxanthine phosphoribosyltransferase 1; *IL* interleukin; *iNOS* inducible nitric oxide synthase

### Protein extraction analysis by western blot

Cells were stimulated as previously indicated for 24 h and intracellular proteins were extracted employing Tris–HCl buffer pH 7.5 with protease inhibitor cocktail and phenylmethylsulfonyl fluoride (all from Sigma-Aldrich). Thereafter, proteins were separated by SDS-PAGE electrophoresis as previously described [46]. Once separated, proteins were transferred to membranes and then incubated with the following antibodies: anti-human rabbit Nrf-2 (1:1000) (SantaCruz Biotechnology) and HO-1 (1:1000) (Enzo Life Sciences, New York, USA) overnight at 4 °C and anti-human mouse tubulin (1:1000) (Sigma-Aldrich) 1 h at room temperature. Anti-rabbit or mouse secondary antibody (1:1000, Dako, Germany) and ECL chemiluminescent substrate (Millipore, USA) were used to detect antigen–antibody binding. Protein bands were quantified by densitometry with the ImageQ image processing software (http://imagej.nih.gov/). All protein band intensities were normalized to the tubulin band intensity for the same sample.

### Immunohistochemistry

Immunohistochemical studies were performed in sections of paraffin-embedded samples of biopsies of synovial tissue from OA and OA-DB patients. Immunohistochemical assays were also performed in GYY-4137 or vehicle-intraarticular injected rats under an experimental OA model performed in a previous study [63]. Samples were deparaffinised, cleared with xylene, and hydrated in a series of increasing grade alcohol. Heat-mediated antigen retrieval was performed in ethylenediaminetetra-acetic acid (EDTA) buffer (pH 9.0; Dako, Agilent Technologies Spain S.L., Barcelona, Spain) for CBS and CSE detection or citrate buffer (pH 6.1; Dako) for CD68 detection. Thereafter, peroxidase blocking solution (Dako) was used to block endogenous peroxidase activity and sections were additionally incubated for 30 min with PBS containing BSA 1% to block non-specific antibody binding when immunohistochemistry of CD68 was performed. Then, slides were washed with phosphate buffer solution and incubated with primary antibodies against CBS (1:200) and CSE (1:300) (Abcam) for human samples or CD68 (1:100; Santa Cruz Biotechnology, Heidelberg, Germany) for rat samples. The antigen–antibody reaction was detected with the anti-rabbit/mouse or anti-goat peroxidase-conjugated secondary antibodies and visualized by DAB DAKO REAL EnVision Detection Kit (Dako). Sections were counterstained with haematoxylin. Slides were observed in an Olympus Dx61 optical microscope (Olympus España S.A.U., Barcelona, Spain). Staining intensity was quantified using ImageJ software.

### Flow cytometry

Protein expression of CD86 and CD206 were analysed by flow cytometry using a FACSCalibur cytometer (Becton Dickinson). TPH-1 cells were differentiated and stimulated as previously indicated. Then, cells were collected, washed with phosphate buffered saline (PBS), and incubated with fluorescein isothiocyanate–labeled anti–CD206 (Biolegend, San Diego, USA) and phycoerythrin-labeled anti–CD86 (Biolegend) for 30 min at 4 °C. After washing, fluorescence intensity was analysed by flow cytometry in the fluorescence channel 1 (CD206) or fluorescence channel 2 (CD86), and expressed as percentage of positive cells per each protein.

### ELISA

TPH-1 cells were treated as previously indicated for 24 h. The levels of IL-6 and IL-8 in culture supernatants harvested after stimulation were determined using commercially available DuoSet ELISA kits for IL-6 and IL-8 (Bio-Techne R&D Systems, Madrid, Spain) according to the recommendations of the manufacturers. Data are expressed as picograms released per mL. The working range was between 9.38 and 600 pg/mL for IL‐6 and 31.25 to 2,000 pg/ml for IL-8.

### Cell proliferation assay

Differentiated TPH-1 cells were treated as indicated for 24 h. The BrdU Cell Proliferation Assay Kit (Cell Signaling Technology) was employed to evaluate cell proliferation, which evaluates the incorporation of 5-bromo-2′-deoxyuridine (BrdU), a thymidine analog, according to the manufacturer’s instructions.

### Statistical analysis

Data are expressed as the mean from «n» independent experiments (*n* = number of patients or assays) ± standard error of the mean (SEM) or as representative results, as indicated. Results were analysed using the GraphPad PRISM version 8 statistical software (La Jolla, CA, USA). Differences between experimental conditions were determined by non-parametric Wilcoxon test (paired comparison) and Mann–Whitney test (comparison between independent samples). *p* ≤ 0.05 was considered statistically significant.

## Results

### Expression of main H_2_S-producing enzymes in synovial tissue from DB and non-DB OA patients

**Fig. 1:**
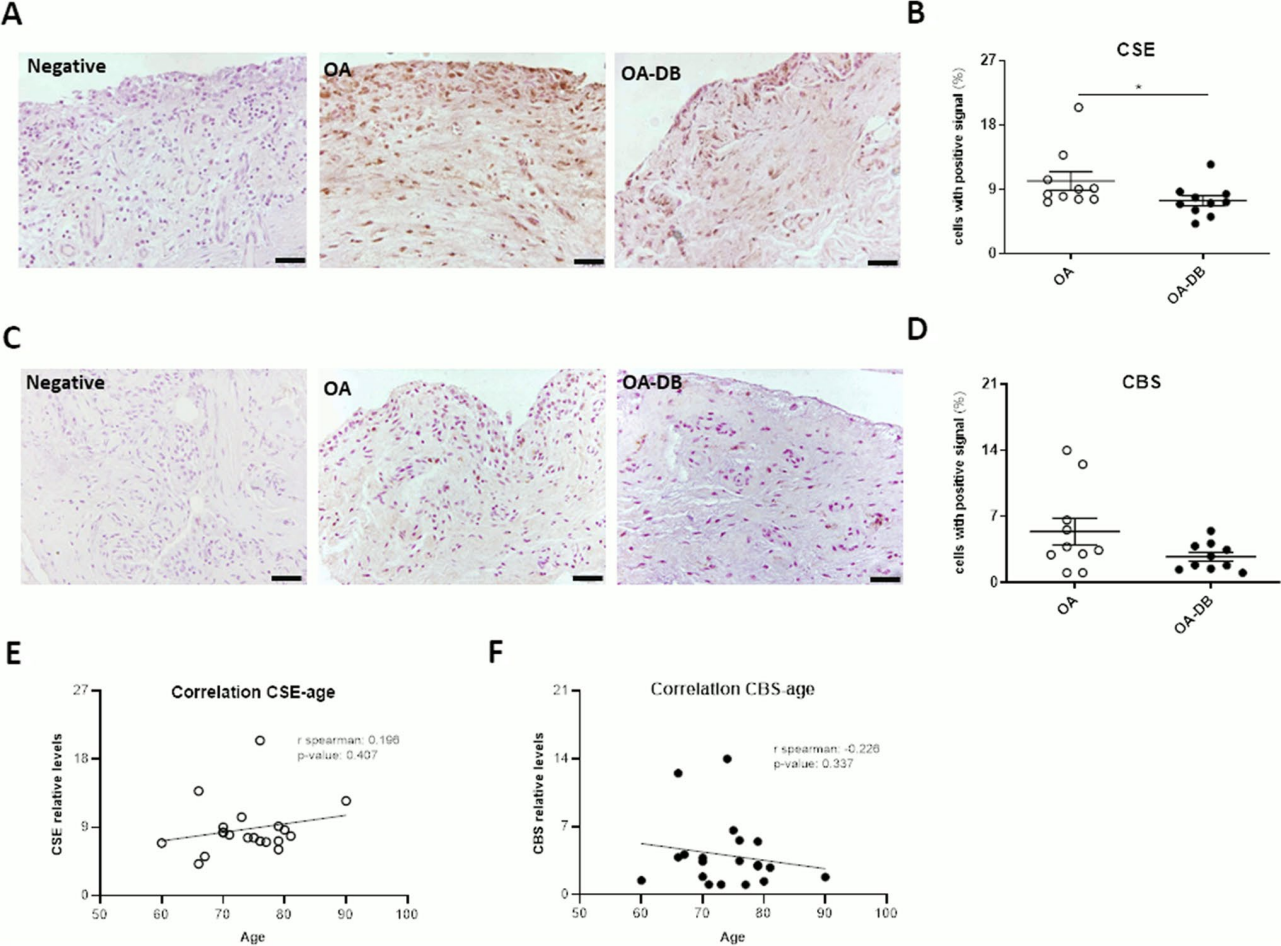
Analysis of H_2_S-synthetizing enzymes expression in synovial tissues. Representative images of CSE (**A**) and CBS (**C**) immunohistochemistry in OA patients with diabetes (OA-DB) or without diabetes (OA). Negative, samples incubated without primary antibody. Images were taken at × 20. Scale bar represents 50 μm. Quantitative analysis of CSE (**B**) and CBS (**D**) positive cells. Correlation analysis of CSE (**E**) or CBS (**F**) expression and age. Values are mean ± SEM (*n* = 10 independent patient for each condition). * *p* ≤ 0.05 vs. OA group

Biopsies of synovial tissue from OA patients with or without DB diagnosis were employed to analyse the expression of CSE and CBS in the tissue. As shown in the Fig. 1, protein levels of both enzymes were lower in synovium from DB-OA patients than in those from OA patients, only achieving significant differences for CSE expression (Fig. 1A and B). In addition, we discarded that age could influence on expression of CSE or CBS independently of having DB (Fig. 1E and F).

### Expression of main H_2_S-producing enzymes and phenotypic characterization of differentiated macrophages under high glucose environment

**Fig. 2:**
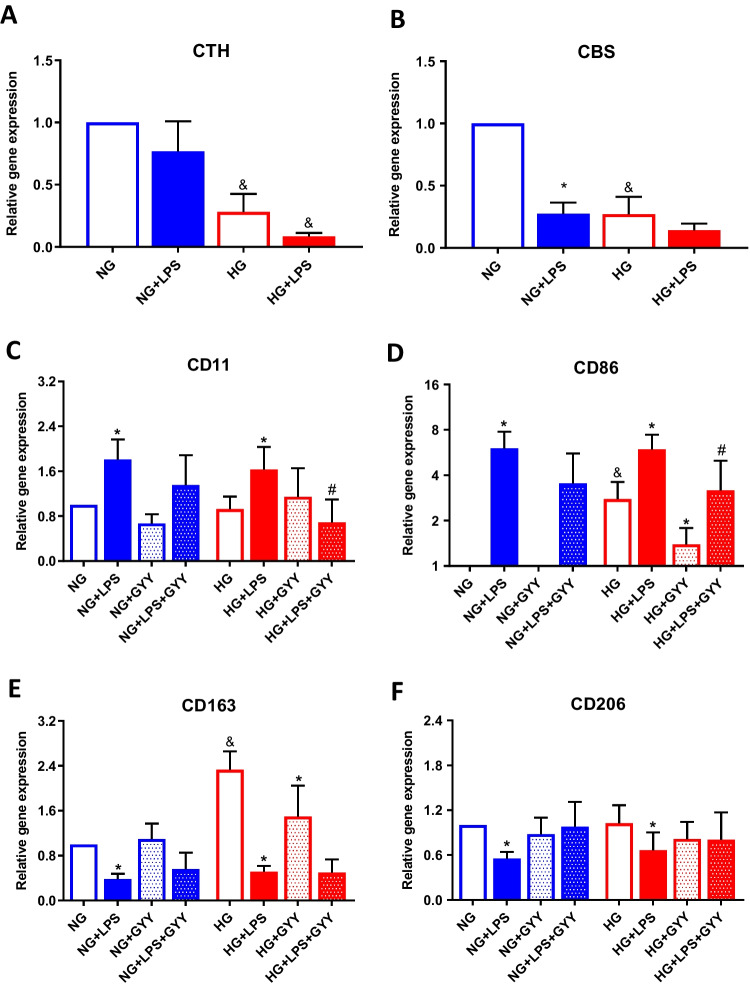
Analysis of expression of phenotypic markers for macrophages. Gene expression of CSE (**A**), CBS (**B**), CD11c (**C**), CD86 (**D**), CD163 (**E**), and CD206 (**F**) in TPH-1 cells incubated with high glucose (HG) or normal glucose (NG) in the presence or absence of LPS and co-treated with the slow-releasing H_2_S donor GYY-4137 (GYY) when indicated. Values are mean ± SEM (*n* = 5 independent experiments). * *p* ≤ 0.05 vs. respective basal condition; ^&^ *p* ≤ 0.05 vs. respective condition incubated with normal glucose; ^#^ *p* ≤ 0.05 vs. respective condition stimulated with LPS

Cell line of monocytes, TPH-1, was differentiated into macrophages as previously indicated and stimulated with LPS, an inductor of M1-like macrophage, for 24 h under normal or high concentration of glucose in order to evaluate if glucose stress could modulate the expression of H_2_S-producing enzymes in these cells. First, we analysed the gene expression of CBS and CSE, observing that incubation with high glucose elicited a reduction of levels of both enzymes (Fig. 2). LPS stimulation also reduced CBS and CSE gene expression in comparison with non-treated cells under both normal or high levels of glucose. Remarkably, the reduction of CSE expression induced by LPS was significantly higher when cells were incubated in high glucose (Fig. 2B).

To investigate whether addition of an exogenous source of H_2_S could modulate the polarization of macrophages under normal or high levels of glucose, untreated or LPS-activated macrophages were co-treated with the slow release H_2_S donor, GYY-4136. Thereafter, gene expression of classic markers of M1- and M2-like macrophages were evaluated. As shown in Fig. 2, gene expression of CD86 (Fig. 2C) and CD11 (Fig. 2D) were significantly upregulated by LPS stimulation under both normal and high glucose incubation (*p ≤ 0.05 vs. respective basal condition). Whereas LPS treatment downregulated CD163 (Fig. 2E) and CD206 (Fig. 2F) gene levels in comparation with basal conditions (*p ≤ 0.05). Moreover, incubation of non-treated macrophages with high levels of glucose strongly increased the expression of CD86 and CD163 (^&^*p* ≤ 0.05 vs. low glucose incubation). Interestingly, co-treatment with GYY-4137 inhibited the upregulated levels of CD86 observed after incubation of cells with high levels of glucose (*p ≤ 0.05 vs. respective basal condition), as well as the CD11 and CD86 gene expression elicited by LPS under high glucose conditions (^#^*p* ≤ 0.05 vs. respective condition stimulated with LPS).

**Fig. 3:**
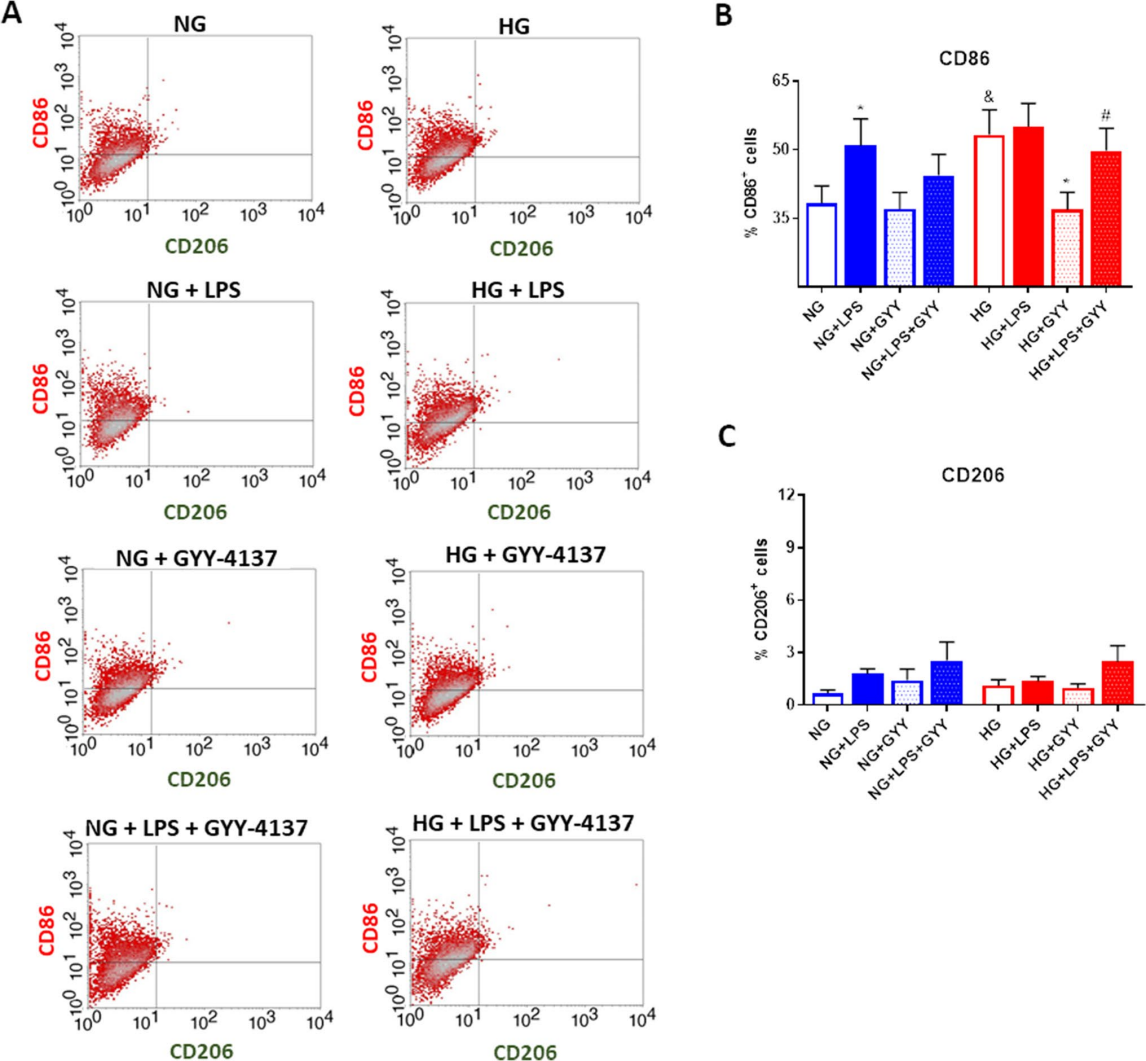
Analysis of protein expression of phenotypic markers for macrophages. **A** Representative images of analysis of CD206 (x axis) and CD86 (y axis) expression by flow cytometry in TPH-1 cells incubated with high glucose (HG) or normal glucose (NG) in the presence or absence of LPS and co-treated with the slow-releasing H_2_S donor GYY-4137 (GYY). Quantitative analysis of CD86 (**B**) and CD206 (**C**) protein expression. Values are mean ± SEM (*n* = 6 independent experiments). * *p* ≤ 0.05 vs. respective basal condition; ^&^ *p* ≤ 0.05 vs. respective condition incubated with normal glucose; ^#^ *p* ≤ 0.05 vs. respective condition stimulated with LPS

Results obtained were afterwards confirmed through analysis of protein levels of CD86 and CD206 by flow cytometry (Fig. 3A). As previously observed at gene level, treatment with LPS (*p ≤ 0.05) or incubation with high levels of glucose (^&^*p* ≤ 0.05) triggered an augmentation of protein expression of CD86 compared with that detected in non-treated cells under normal glucose environment (Fig. 3B). It was also particularly noteworthy that GYY-4137 treatment significantly attenuated CD86 expression induced by high levels of glucose in both the absence (*p ≤ 0.05) or presence of LPS (^#^*p* ≤ 0.05) (Fig. 3B). Conversely, we failed to observe any significant modulation in CD206 protein levels under the different experimental conditions tested (Fig. 3C), obtaining similar results than detected when the mRNA expression was analysed (Fig. 2E).

### Proliferation rate and pro-Inflammatory activation to LPS in differentiated macrophages under high glucose environment

**Fig. 4:**
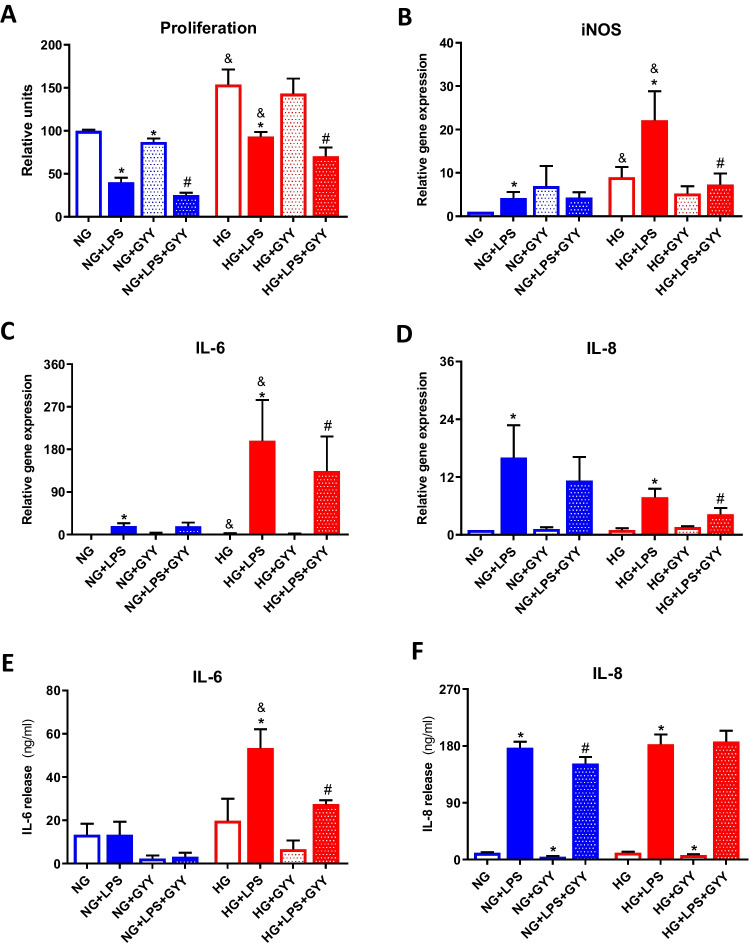
Proliferation rate and expression and release of pro-inflammatory mediators. Proliferation rate (**A**) and gene expression of iNOS (**B**), IL-6 (**C**), and IL-8 (**D**) in TPH-1 cells incubated with high glucose (HG) or normal glucose (NG) in the presence or absence of LPS and co-treated with the slow-releasing H_2_S donor GYY-4137 (GYY). Release of IL-6 (**E**) and IL-8 (**F**) measured by ELISA. Values are mean ± SEM (*n* = 5 independent experiments). * *p* ≤ 0.05 vs. respective basal condition; ^&^ *p* ≤ 0.05 vs. respective condition incubated with normal glucose; ^#^ *p* ≤ 0.05 vs. respective condition stimulated with LPS

In order to further characterize the macrophage phenotype, proliferation rate was also evaluated. As shown in Fig. 4a, LPS stimulation notably decreased levels of proliferation in cells incubated in both normal or high levels of glucose (*p ≤ 0.05). Likewise, incubation with high glucose upregulated proliferation rate in non- and LPS-treated cells under normal levels of glucose (^&^*p* ≤ 0.05). Finally, we observed that co-treatment with GYY-4137 diminished proliferation levels detected in basal (*p ≤ 0.05) and LPS (^#^*p* ≤ 0.05) stimulated macrophages.

Considering that LPS is known to be a potent inductor of inflammatory responses associated to M1-like macrophage activation [14, 17], we evaluated the gene expression of inducible nitric oxide synthase (iNOS), IL-6, and IL-8 in PMA-differentiated TPH-1 cells stimulated as previously indicated for 24 h. As expected, LPS stimulation upregulated gene levels of all pro-inflammatory mediators tested, under both normal and high glucose conditions (*p ≤ 0.05) (Fig. 4B-D). Interestingly, macrophages incubated with high glucose showed a higher LPS-induced expression of IL-6 and iNOS than those cells cultured under normal levels of glucose (^&^*p* ≤ 0.05). When cells were co-treated with the slow-releasing H_2_S donor, GYY-4137, we observed a significant attenuation in mRNA levels of all mediators in LPS treated macrophages under high glucose environment (^#^*p* ≤ 0.05). Besides, these results were further confirmed at protein level by ELISA (Fig. 4E-F). As previously observed, release of IL-6 induced by LPS was augmented in cells incubated with high glucose in comparison with those under normal glucose (^&^*p* ≤ 0.05), being this response attenuated by co-treatment with GYY-4137 (^#^*p* ≤ 0.05) (Fig. 4E). Nonetheless, we failed to observe any relevant impact of incubation with normal or high glucose nor GYY-4137 co-treatment on IL-8 production elicited by LPS (Fig. 4F).

### Involvement of Nrf-2/HO-1 axis and HIF-1α in high glucose-mediated activation of differentiated macrophages

**Fig. 5:**
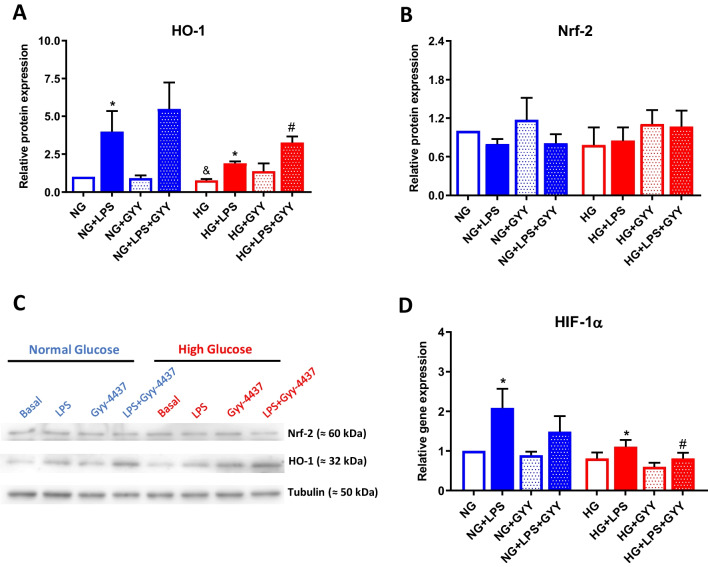
Analysis of HO-1, Nrf-2, and HIF-1α expression. Protein expression of HO-1 (**A**) and Nrf-2 (**B**) in TPH-1 cells incubated with high glucose (HG) or normal glucose (NG) in the presence or absence of LPS and co-treated with the slow-releasing H_2_S donor GYY-4137 (GYY). **C** Representatives images of one experiment evaluating protein expression of Nrf-2 and HO-1 by western blot. Tubulin levels were employed as load control. **D** Gene expression of HIF-1α. Values are mean ± SEM (*n* = 5 independent experiments). * *p* ≤ 0.05 vs. respective basal condition. ^&^ *p* ≤ 0.05 vs. respective condition incubated with normal glucose

Nrf-2/HO-1 axis is an important antioxidant system that has been described to be impaired in OA and DB patients and play a pivotal role in their pathogenesis [46, 64]. We first analysed the protein expression of HO-1 in differentiated TPH-1 cells stimulated with LPS for 24 h under different glucose conditions. As shown in Fig. 5A and C, LPS stimulation upregulated HO-1 protein levels under both normal and high glucose incubation (*p ≤ 0.05), also observing that cells under high glucose environment showed reduced expression of HO-1 compared to those incubated with normal levels of glucose (^&^*p* ≤ 0.05). Interestingly, it was also noticed that co-treatment with GYY-4137 further enhanced HO-1 expression induced by LPS (#*p* ≤ 0.05). However, non-significant modulation of Nrf-2 protein levels was detected in any of tested conditions (Fig. 5B and C). Moreover, we analysed the expression of transcription factor hypoxia-inducible factor-1 alpha (HIF-1α), a key regulator of immune responses [40]. LPS stimulation upregulated HIF-1 α expression (*p ≤ 0.05), this effect appeared to be lower in those macrophages exposed to high glucose levels, although differences detected between conditions failed to reach statistical significance (Fig. 5D). Finally, we observed that the addition of GYY-4137 modulated HIF-1 levels induced by LPS, achieving a significant reduction in cells incubated with high glucose (^#^*p* ≤ 0.05) (Fig. 5D).

### Modulation of CD68^+^ macrophage abundance in synovial tissue from mice joints injected intraarticularly with GYY-4137, a slow donor of H_2_S

**Fig. 6:**
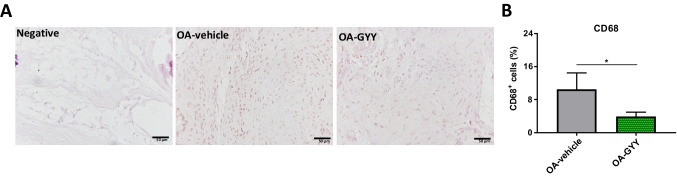
Analysis of CD68^+^ cells abundance in synovial tissues. **A** Representative images of CD68 immunohistochemistry in synovial tissue from rats undergoing surgical OA injected with intraarticularly saline (OA-vehicle) or GYY-4137 (OA-GYY). Negative, samples incubated without primary antibody. Images were taken at × 20. Scale bar represents 50 μm. **B** Quantitative analysis of CD68^+^ cells. Values are mean ± SEM (*n* = 3 independent rats for each condition). * *p* ≤ 0.05 vs. OA-vehicle group

To confirm our findings suggesting upregulation of H_2_S as modulator of macrophage activation and infiltration, we evaluated the effect of intraarticular injection of GYY-4137 on macrophage abundance in synovial tissue from a previously assayed model of experimental OA in rats by destabilization of medial meniscus [63]. Immunohistochemistry of CD68, a known marker of macrophages [20, 21], were hence performed in histological sections of joints from saline- and GYY-4137-treated rats undergoing a surgical OA. As shown in Fig. 6, synovial tissues from rats injected intraarticularly with the slow-releasing H_2_S donor presented lower number of CD68^+^ cells than in the synovium obtained from saline-injected rats.

## Discussion

A growing number of evidence indicate that inflammation contributes to OA development and thus, innate immune system has a pivotal role in this pathology [50, 52, 54]. Macrophages, effector cells in innate immunity, are involved in different processes taking place in the joint, including both tissue regeneration and inflammation [11]. Macrophage polarization is critical to define the function that these cells is playing in the joint, being recently suggested that H_2_S-mediated pathways could influence in the decision of macrophage fate [59]. To further elucidate this issue and determine the role of H_2_S on synovial inflammatory phenotype associated to OA patients with diabetes, in the present study, we observed for the first time to our knowledge that the expression of H_2_S synthesizing enzymes is reduced in synovial tissue from this subset of OA patients. This event appears to be mimicked in LPS-activated macrophages when exposed *in vitro* to glucose stress. Likewise, our results suggest that reduced production of H_2_S in OA patients with diabetes could favour pro-inflammatory activation of macrophage since treatment with a slow-releasing H2S donor, GYY-4137, modulates macrophage phenotype and synthesis of pro-inflammatory mediators induced by LPS in the presence of high levels of glucose. Besides, *in vivo* experiments show that intraarticular administration of GYY-4137 attenuates synovial infiltration of macrophages in a surgical model of OA, reinforcing thus our findings.

Biosynthesis of H_2_S is mainly controlled by catabolic activity of two enzymes: CBS and CSE [12, 47]. Defects in their expression or activity have been associated to different pathologies [22, 56, 65]. In this study, we observed that levels of CSE, and CBS to a lesser extent, were reduced in synovial tissue from OA patients with type II diabetes. In agreement, previous studies in our group demonstrated that H_2_S levels and biosynthesis are defective in sera and cartilage from OA patients, and specially in those also harbouring metabolic disorders like type II diabetes [4, 46]. Hence, reduced production of H_2_S has been linked to lower expression of CSE, CBS, and MPST in the joint. Alteration of H_2_S levels is now considered a key event in the pathogenesis of diabetes and associated complications [46, 61, 69] as for instance, a downregulation in CSE and CBS expression favours progression of diabetic cardiomyopathy [58]. In relation, CSE levels and H_2_S content are significantly reduced in high glucose-treated cardiomyocytes [25], suggesting that activation of glucotoxicity-associated pathways could be a mechanism underlying the H_2_S dysregulation observed in diabetes. In our study, macrophages treated with high glucose showed reduced gene expression of CSE and CBS. Likewise, similar results were previously observed in IL-1β-activated chondrocytes incubated with high glucose [46].

A growing number of *in vivo* and *in vitro* studies indicates that induction of H_2_S production in the joint has protective effects against pathological pathways taking place in rheumatic diseases like OA [5, 37, 46, 63]. In order to further elucidate these findings and evaluate whether H_2_S dysregulation participates in synovial inflammation associated to macrophages observed in metabolic OA [9], we analysed *in vitro* effect of high glucose exposure on macrophage polarization toward an inflammatory M1-like phenotype. Besides, we examined if the administration of a slow-releasing H_2_S donor could modulate these responses.

Our results showed that LPS-activated macrophages exposed to glucose stress exhibit a more patent pro-inflammatory phenotype, characterized by higher expression of membrane proteins CD86 and CD11c, both classically associated to M1 phenotype [49, 79], and iNOS, enzyme involved in NO synthesis from l-arginine that have been linked to inflammatory processes [21, 55]. Whereas CD206 expression, marker of M2 phenotype [49, 79], remained unchanged after treatment. Interestingly, we failed to detect the expression of M2-like macrophage indicator argininase-1 (data not shown), a pivotal enzyme involved in the regulation of the immune response, mainly through its competition with iNOS for the same substrate, l-arginine, resulting in the protection of tissues against inflammation [39]. Conversely, activated cells under high glucose exposure also showed increased expression of CD163, a protein membrane commonly linked to M2 macrophages [79]. Nonetheless, different phenotypes observed in macrophages generally failed to simply adjust to classic categories M1 and M2, but there are intermediate phenotypes. CD163 is a scavenger receptor specifically expressed in monocytes and macrophages that has been described to be proteolytically cleaved from the plasma membrane in response to oxidative stress and LPS stimulation, generating a soluble CD163 form (sCD163) [57]. Noteworthy, sCD163 has been described to be a predictive marker for development of type 2 diabetes and related complications [34, 53], as well as associated with insulin resistance and lipid metabolism [42, 53, 62]. Greisen et al. observed that macrophage-derived sCD163 is a reliable marker of disease activity and radiographic progression in early RA patients [15]. Therefore, studies are warranted to determine whether sCD163 levels are altered in the metabolic phenotype of OA.

Macrophages activated by pro-catabolic molecules like LPS produce a great number of pro-inflammatory mediators including NO, PGE_2_, TNF-α, IL-6, and IL-8, as well as regulatory enzymes (e.g., iNOS and COX-2) [7, 33, 80]. Short- and long-term incubation with high glucose sensitizes macrophages to cytokine stimulation [43]. Consistently, we also observed that macrophages exposed to high glucose presented a higher expression and release of IL-6 and iNOS, as previously mentioned, than those cells under normal levels of glucose. However, IL-8 expression and production were inconsistently modulated. Similarly, our group and others have previously detected that joint cells undergoing glucose stress showed a higher response to catabolic stimuli, increasing the expression of pro-inflammatory mediators [23, 64]. Since growing number of evidence indicates that H_2_S could play a role in macrophage polarization towards an anti-inflammatory phenotype [59, 78], we tested its effect on the profile of macrophage induced by glucose stress. Hence, the administration of a slow-releasing H2S donor, GYY-4137, downregulated the expression of M1 markers (i.e., iNOS, CD11c, and CD86) elicited by LPS under our *in vitro* approach of hyperglycaemia. In agreement with these findings, different studies have observed that exogenous administration of H_2_S attenuates M1 polarization, including reduction of CD86 [24], CD11c [60], and iNOS [77] expression. H_2_S has also been indicated to lead to the polarization of macrophages from M1 to M2 phenotype [77], increasing the expression of M2 markers like CD206 [13, 77] and CD163 [1]. In our study, GYY-4137 co-treatment failed to recover the reduction in gene expression of CD206 triggered by LPS, whereas protein levels were barely detected. While CD163 overexpression elicited by glucose stress was diminished by GYY-4137, although, as previously commented, the biological significance of CD163 modulation is needed to be determined in future studies. In addition, induction of H_2_S production attenuated IL-8 and IL-6 gene expression under high glucose, as well as the release of IL-6 but not IL-8. Previous studies from our group and others showed a similar anti-inflammatory effect of H_2_S donors in joint cells [5, 26, 46], also inhibiting LPS-induced inflammation in macrophages [73, 77, 80]. All together, these findings suggested that H_2_S inhibits cell polarization towards M1-like macrophages induced by glucose stress, but more studies are warranted to further define phenotypic outcome after H_2_S treatment.

Proliferation rate of macrophages was also evaluated in our *in vitro* model of hyperglycaemia, observing that high glucose exposure induced an increment in macrophage proliferation, a process that is linked to diabetes and its related pathologies [3, 27, 35]. By contrast, LPS significantly reduced cell proliferation as other authors have already detected [10, 28]. It has been described that pro-inflammatory stimulation suppresses Myc-dependent cell proliferation while promoting a HIF1α-dependent transcriptional program to maintain heightened glycolysis in M1 macrophages [28]. In this respect and in accordance with other studies [38], LPS increased HIF-1α expression in our study, whereas the addition of a H_2_S-releasing donor attenuated cell proliferation as other authors had previously observed [80] and inhibited HIF-1α upregulation mediated by LPS. Taken together, our results suggest the involvement of HIF-α in LPS effect on macrophage proliferation, which could be modulated by the induction of H_2_S production.

H_2_S exerts its actions via activation or repression of different signalling pathways [59, 69]. As observed in previous studies from our group and others, the antioxidant pathway Nrf-2/HO-1 is activated by H_2_S, being involved in some of the anti-inflammatory effects of the gas in diabetes-associated pathologies including OA-DB [46, 69]. In this regard, a decline of Nrf-2/HO-1 system has been detected in these patients and suggested to underlie the link between both pathologies [46, 64]. Different studies have described that activation of Nrf2 and HO-1 expression could block M1 stimuli-induced production of pro-inflammatory mediators like LPS, and shift the polarization of macrophages toward M2 type [68, 71]. Likewise, H_2_S could regulate macrophage phenotype through activation of this pathway [32, 59]. In our study, treatment with H_2_S-releasing donor failed to modulate Nrf-2 protein levels, but upregulated HO-1 expression. The regulation of Nrf-2 activity can occur at transcription, translation, and post-translational levels [45], the latter controlling Nrf-2 translocation into the nucleus and binding to promoter sequences of its target genes, like HO-1. Hence, this regulation should be evaluated in future assays. In addition, a number of transcription factors such as activator protein-1, nuclear factor- kappaB, or mentioned Nrf-2, and some of the upstream kinases have been identified as main regulators of HO-1 gene induction [48], and thus H_2_S may promote the expression of this enzyme via activation of Nrf-2-independent pathways.

Administration of exogenous sources of H_2_S has also been described to regulate macrophage infiltration, alleviating inflammation and tissue damage in *in vivo* models of diabetes-related complications as well as other pathologies [75, 76, 80]. In our study, intraarticular injection of slow-releasing H_2_S donor reduced macrophage abundance in synovial tissue in an *in vivo* surgical model of OA. Likewise, we had previously observed that joints under this treatment also showed lower pain and cartilage damage as well as improved joint function [63]. Similarly, a recent study demonstrated in an *in vivo* murine model of rheumatoid arthritis that a simple block polymer was able to modulate macrophage polarization and mitigate synovial inflammation, osteoporosis, and clinical symptoms, which was attributed to its H_2_S-releasing and NO-scavenging properties [13].

Overall, the results observed in this study indicate that synovial tissue from OA patients with diabetes show a reduced expression of H_2_S-synthesizing enzymes, an event that appears to be mimicked *in vitro* in macrophages incubated with high glucose levels. Likewise, glucose stress favours macrophage polarization towards a pro-inflammatory phenotype since higher expression of M1 markers (i.e., CD86, CD11, iNOS, and IL-6) and greater proliferation rate were detected *in vitro* in cells exposed to high glucose. The co-treatment with a slow-releasing H_2_S donor, GYY-4137, modulated macrophage phenotype, reducing the expression of pro-inflammatory mediators and lowering the cell proliferation. In addition, the intraarticular administration of a H_2_S donor attenuated synovial abundance of CD68^+^ cells, mainly macrophages, in an *in vivo* model of OA. Therefore, our study seems to reinforce the key role of H_2_S in OA and specifically its metabolic phenotype, as well as sheds light on its effects on the polarity of synovial macrophages, opening consequently new therapeutic perspectives in the management of this pathology.

## Data Availability

The data used to support the findings of this study are contained within the article. Raw data are available from the corresponding author upon request.
